# Seasonal Variation in Nutritional and Chemical Profiles of Wild *Opuntia ficus-indica* Fruits

**DOI:** 10.3390/plants14030409

**Published:** 2025-01-30

**Authors:** Ana O. S. Jorge, Anabela S. G. Costa, Diana Melo Ferreira, Maria Beatriz P. P. Oliveira

**Affiliations:** LAQV@REQUIMTE, Faculdade de Farmácia, Universidade do Porto, R. Jorge Viterbo Ferreira 228, 4050-313 Porto, Portugal

**Keywords:** *Opuntia*, prickly-pear cactus, seasonal variation, nutritional composition, *Opuntia ficus-indica*

## Abstract

*Opuntia*, a cactus known for its edible fruit, produces both summer and winter fruit with distinct nutritional profiles. This study, using samples from the same plant in Northeast Portugal, revealed that winter seeds contain 2.4 times more fructose and 1.8 times more total sugars than summer seeds. Winter samples also showed a 2.3-fold increase in α-tocopherol content (11.69 mg/kg vs. 5.17 mg/kg in summer) and higher moisture (seed moisture: 54.96% vs. 49.52%). The peel consistently displayed the highest antioxidant activity, with summer samples generally having stronger antioxidant status. Interestingly, no statistical significance was found for the antioxidant activity or bioactive in the seed, both remaining constant between seasons. The fatty acid composition remained largely consistent, with linoleic acid as the dominant component (57–59%), though winter oil showed slightly higher saturation. Nutritional content was higher in winter samples, while bioactive constituents were more abundant in summer, and fatty acid composition remained stable across both seasons. These results emphasize the impact of seasonality on the nutritional and bioactive properties of *Opuntia* fruits.

## 1. Introduction

*Opuntia ficus-indica* (L.) Mill, known as prickly-pear cactus or barbary fig, is a fruit-bearing cactus native to Mexico and is now found worldwide due to its resilience and adaptability to various environments. It is invasive in Europe and Africa and a profitable crop in low-water areas. Lower water availability due to climate change effectively increases this plant’s territory as it flourishes in arid and semiarid habitats [1]. *Opuntia* thrives in high temperatures, high CO_2_ levels, and low rainfall while showing high biomass productivity and extensive root growth [2]. The cactus exhibits Crassulacean Acid Metabolism (CAM) photosynthesis, allowing it to fix carbon dioxide during the night to reduce water loss during the day [1]. The ideal temperature for *Opuntia* root growth is reported to be between 27 °C and 30 °C, while low temperatures (−16 °C) can damage the plant [3]. Temperatures of 2–6 °C are well tolerated by the plant, making it suitable to inhabit colder regions like southern Europe [4]. The most limiting factor to *Opuntia* growth in these regions is the low temperatures in winter [5].

Human consumption of this plant has been linked with various health benefits. Its high content of digestible carbohydrates and antioxidant dietary fiber [6], the capacity to behave as radical scavengers [7], its high content of minerals, antioxidants, and vitamin C [8], and its seed oil particularly rich in PUFAs/linoleic acid are some compositional features that bring benefits to conditions such as coronary heart disease and rheumatoid arthritis. An example of a direct action of consuming prickly pear fruits is the protective ability against ethanol-induced stomach ulcers [9]. Extracts have been proven effective in lowering cholesterol [10] and preventing type-II diabetes [11], and aqueous extracts are reported to inhibit in vitro growth of cancer cells [12]. The fruit has high levels of ascorbic acid, vitamin E, carotenoids, betalains, fiber, glucose, and fructose. The plant is also a natural source of antioxidants, guarding against cellular damage and being considered a functional food by many [13]. Polyphenols are primarily responsible for their antioxidant properties. They include phenolic acids and flavonols, which vary significantly across different plant parts and cultivars [14]. Moreover, the cactus is a good source of vitamin C, the fruit containing 0.26–0.49 mg/g fresh weight of ascorbic acid [15]. For this, it has seen wide use in traditional medicine. In Turkish folk medicine, the fruits of *Opuntia ficus-indica* have been used to treat anxiety, burns, bronchial asthma, constipation, kidney stones, and rheumatic pains [16]. Moreover, there are registers pointing to its use for anti-inflammatory effects, hypoglycemic effects, and neuroprotective effects [17].

Betalains are unique bioactive pigments found only in the order Caryophyllales. They are responsible for the characteristic red/orange color of the prickly-pear fruit. Betanin and indicaxanthin, the primary betalains in *Opuntia ficus-indica*, exhibit strong radical scavenging abilities, outperforming standard antioxidants like Trolox in certain assays [18]. The antioxidant capacity of *Opuntia ficus-indica* has been shown to be highly correlated with its betalain content [19].

*Opuntia ficus-indica* is a perennial succulent that forms a bushy or tree-like structure. Like most cacti, the stems are modified into flattened, fleshy, and paddle-shaped segments called cladodes, which are covered with a thick waxy cuticle to minimize water loss [1]. Unlike some cacti, *Opuntia ficus-indica* has reduced or absent spines, making it less harmful to handle. The fruit is an oblong or pear-shaped berry, commonly known as a prickly pear, with the outer skin covered with glochids, while the inner pulp is juicy, sweet, and contains numerous hard seeds [1]. Most varieties follow the same development throughout the year. Flowering occurs in spring, usually in May, followed by fruit development harvested in summer. Some plants also present a second out-of-season fruit production in winter. It is important to note that the ability of *Opuntia* to produce fruits in winter may vary, depending on the specific climate conditions and cultivars, as it is found to be dependent on abiotic factors such as nitrogen present in the soil [14] and the removal of cladodes and flowers during the primary season [15].

In the northeast region of Portugal, a colony of wild *Opuntia* has been growing over the past few decades and probably migrated from Spain. The northeast region, particularly Trás-os-Montes, is characterized by a Mediterranean climate with continental influences. This area experiences hot dry summers and cold winters, with significant temperature fluctuations between day and night [20]. Rainfall is relatively low, averaging between 400 and 800 mm annually, with most precipitation occurring during the autumn and winter months. Summers are typically arid, which aligns well with the drought-resistant nature of the *Opuntia* genus. Furthermore, the soils in Trás-os-Montes are often poor in organic matter and well-drained, conditions that are suitable for the hardy *Opuntia* species [20]. Due to the lack of studies regarding wild plant varieties, a nutritional study into this variety of summer and winter fruit was conducted, highlighting their differences. This research aimed to provide a deeper understanding of the seasonal variations in the nutritional and bioactive compound profiles of *Opuntia ficus-indica* fruits, with a focus on how environmental factors influence their composition. The scientific relevance lies in the potential use of wild varieties as a sustainable source of bioactive compounds and nutrients, which are increasingly valued for their health benefits.

## 2. Methodology

**Figure 1:**
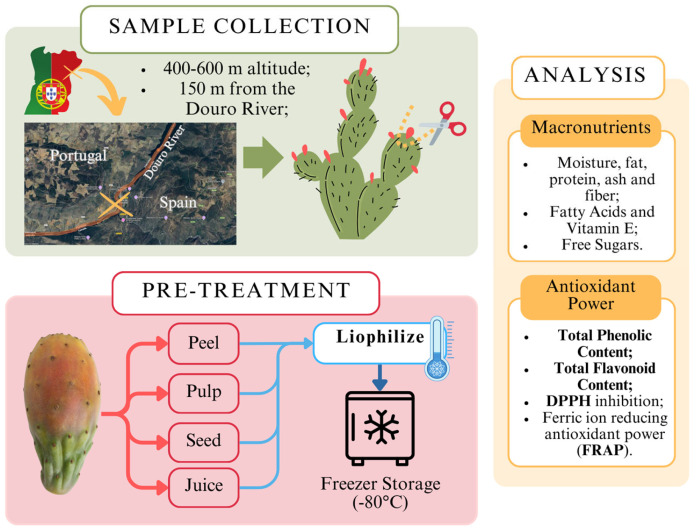
Workflow for cactus fruit (*Opuntia* spp.) collection, pre-treatment, and nutritional analysis.

The outline of the methodology process is briefly illustrated in Figure 1, from the collection of samples, pre-treatment, and analysis process. To ensure a throughout analysis, the fruit samples were separated into 4 different constituent parts, including the juice, the pulp, the seed, and the peel. These parts were analyzed separately. All laboratory procedures were conducted in compliance with standard safety protocols to ensure the safety of personnel and the integrity of the samples.

### 2.1. Sample Gathering and Processing

**Figure 2:**
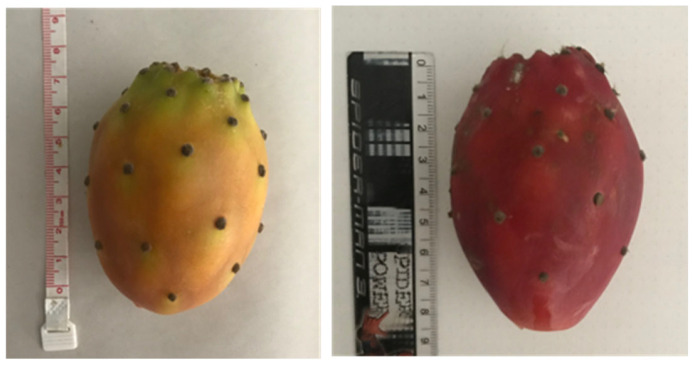
*Opuntia* fruit samples, summer (**left**), and winter (**right**). A different coloration can be noted as the winter fruit is deep red while the summer fruit is orange.

All fruit samples (summer and winter) were collected on the same plant located on the arid slopes near the Saucelle Dam in the municipality of Freixo de Espada à Cinta, Bragança, Portugal, and by the same time of day (late morning) (Coordinates: 40.9956349, −6.8074360). The summer samples were collected in early September 2019, while the winter samples were collected in late December 2019. Samples were transported to the laboratory within less than 24 h and kept in a refrigerated unit. All samples were collected from a single mature *Opuntia ficus-indica* plant, of the orange variety, that did not show any signs of disease or was occulted by other vegetation, with direct sunlight exposure. The samples showed distinct colors but similar sizes (Figure 2).

The winter fruit samples in this study refer to out-of-season winter fruit, belonging to the second stream, which has been described as a phenomenon resulting from natural double flowering under specific environmental conditions [21], whereas the summer fruit samples correspond to the primary natural fruiting cycle, also known as the first stream. Instances of natural double flowering have been recorded in specific regions such as California and Chile, where environmental conditions support prolonged fruiting periods. In these cases, the natural flowering cycle extends the harvest season into late fall or early winter [21]. The fruits obtained in winter in this study could also be the result of a naturally occurring phenomenon of double flowering in the region, as the environmental conditions in the study area may naturally support this extended flowering and fruiting cycle, and the plants received no human intervention. The plant was about 150 m from the Douro River and about 400–600 m altitude [22]. The zone experienced a moderate drought (−2.0–2.99 in the Palmer Drought Severity Index (PDSI)) in the summer of 2019 when the samples were collected and moderate rain (between 2.5 mm and 7.6 mm per hour) during the colder months when the winter 2019 samples were collected [23]. The year 2019 was also a warmer-than-average year, ranking among the warmer years on record in Portugal, consistent with European trends. The region experienced an average temperature of 13.0–13.3 °C, roughly +1.0 to +1.3 °C above the long-term average. Late June to early July 2019 saw a heatwave affecting much of the country, with daily peaks of 35–40 °C [23].

This *Opuntia* wild variety seems to prefer the arid steep slopes as its growing place. This ground is characterized by its lack of growth of other flora, most of it seasonal and only present in winter, when the humidity is increased [20]. This gives *Opuntia* a lack of competition, easing its permanence in the region. The plant seems to prefer slopes directed to the west, perhaps due to increased sun exposure. The proximity of the trees to the Douro River is notable. Although being a specialized arid weather plant, the plant prevalence in this region is higher closer to the river’s margins. The sampling place is characterized by the soil being loose alluvium unconsolidated soil, which sits on top of cambric schist rock [20]. Texturally, the soil is considered a sandy loam, with a moderate proportion of sand (60%), silt (25%), and clay (15%), being slightly acidic (pH 6.3) [20]. It is well-drained with minimal waterlogging potential, being ideal for drought-resistant crops such as *Opuntia*, olives, and almonds [20].

Fruit samples were manually separated into components (pulp, seed, juice, and peel) and homogenized (using the laboratory blender GRINDOMIX GM 200, Retch, Haan, Germany). The peel was obtained by manually removing the outer skin. The pulp was prepared by removing the peel and homogenizing the remaining fruit. The juice was extracted by blending the peeled fruit, leaving the seeds intact, and straining the mixture through a metal sieve. Finally, the seeds were isolated after blending with the juice, ensuring all components were analyzed separately. The samples proceeded to lyophilization (Lyophilizer Freeze Dryer, Telstar, Terrassa, Spain). Lyophilized samples were kept in a dry place, away from sunlight exposure. This methodology is illustrated in Figure 2.

### 2.2. Moisture, Ash, and Macronutrients

Moisture was determined on infrared moisture analyzer, KERN^®^ DBS 60-3 (KERN and SOHN GmbH, Balingen, Germany), using 5.0 ± 0.5 g of sample. The macronutrient tests were carried out using freeze-dried samples and following AOAC methodologies (AOAC 920.15 [24] for ash determination, AOAC 982.08 [25] for protein determination, AOAC 991.36 [26] for fat determination, and AOAC 985.29 [27] for total dietary fiber determination).

### 2.3. Extraction of the Lipidic Fraction

The method for lipid fraction extraction has been employed in previous studies [28]. Briefly, the samples were rigorously weighted to contain 20 mg of total fat and mixed with BHT (75 µL) to prevent oxidation, tocol (50 µL, internal standard), and ethanol (1 mL). After homogenization and the extraction with n-hexane (2 mL), the mixture was vortexed, centrifuged (5 min at 5000 RPM), and reextracted twice with n-hexane. The combined organic phase was dried with anhydrous Na_2_SO_4_, evaporated under a nitrogen stream, resuspended in n-hexane (1 mL), and used for both HPLC analysis (Section 2.4 and Section 2.6) and GC analysis (Section 2.5).

### 2.4. Tocopherol Determination

The HPLC system for Tocopherol determination (Jasco, Tokyo, Japan) was equipped with a DAD MD-2015 multiwavelength diode array detector (Jasco, Tokyo, Japan), a fluorescence detector (FP-2020) (Jasco, Tokyo, Japan) programmed for excitation at 290 nm and emission at 330 nm. An AS-4050 autosampler (Jasco, Tokyo, Japan) and a pump PU-4180 (Jasco, Tokyo, Japan) were used. The capillary column used was Supelcosil™ LC-Si HPLC, 3 µm particle size, L × I.D. 15 cm × 4.6 mm (Merck, Darmstadt, Germany). The eluent was 94:6 n-hexane:1,4-dioxane at a flow rate of 0.7 mL/min. Tocopherols were identified via chromatographic comparison with standards and quantified using a fluorescence detector.

### 2.5. Fatty Acid Profile

The lipid extraction of the samples previously obtained was used to prepare fatty acids for analysis. Fatty acids were further derivatized into methyl esters following ISO 12966-2: 2011 [28]. Briefly, the n-hexane samples were evaporated from the extracts under a nitrogen stream and they were dissolved in dichloromethane (1 mL), mixed with potassium hydroxide methanolic solution (0.5 M, 1 mL), and heated to 100 °C for 10 min. After cooling in ice, boron trifluoride (2 mL) was added, followed by another 30-min heating cycle at 100 °C. Post-cooling, water (2 mL) and n-hexane (2 mL) were added. The organic phase was vortexed and separated via centrifugation (5 min, 3000 RPM), dried with anhydrous Na_2_SO_4_, and injected into a GC-FID system. The GC-FID was equipped with an automatic sampler and injector (AOC-20i Shimadzu, Tokyo, Japan) with a 50:1 split at 250 °C, a silica capillary column CP-Sil 88, 50.0 m × 0.25 mm of internal diameter and 0.20 µm film thickness (Varian, Middelburg, The Netherlands), and a flame ionization detector (Shimadzu, Tokyo, Japan) at 270 °C used helium as the carrier gas (3.0 mL/min). The temperature program was 120 °C for 5 min, then increased by 2 °C/min to 160 °C (held for 2 min) and 220 °C (held for 10 min). Fatty acid methyl esters were identified by comparison with a FAME 37 standard and analyzed based on relative peak areas.

### 2.6. Free Sugar Determination

Free sugars were determined following the previously established protocol [29]. For the sugar extraction, the freeze-dried samples were weighed (250–500 mg depending on the sample’s sugar content, to avoid detector saturation) and 30 mL of water was added, followed by agitation in a vortex mixer for 20 min. The samples were then centrifuged, and 1 mL of the supernatant was filtered and transferred to an HPLC injection vial. This resulted in extracts with a final concentration of 8.33 mg/mL (for the pulp, juice, and peel samples) and 16.66 mg/mL (for the seed). The chromatographic analysis was performed in HPLC equipment, integrated with an evaporative light scattering detector (ELSD), two high-pressure pumps, and an automatic injector. The HPLC system for sugar content determination (Jasco, Tokyo, Japan) was equipped with an automatic sample injector (AS-4050), a pump (PU-4180), an oven (CO-4061), and an evaporative detector with light scattering (ELSD—evaporative light scattering detector) Sedex 80 (SEDERE, Alfortville, France). The compounds were separated in a Shodex column (Asahipak NH2P-50 4E, 4.6 mm ID × 250 mm). An isocratic system with two eluents (water/acetonitrile 1:3) was used and the samples were eluted with a 1 mL/min flux for 20 min at 30 °C. The injection volume was 50 µL.

### 2.7. Determination of Total Phenol Content, Flavonoid Content, and In Vitro Antioxidant Activities

Freeze-dried samples were extracted using a water/ethanol (1:1) solution at 40 °C for 1 h with constant stirring, followed by cooling, filtration, and storage at −20 °C [30]. The total phenolic content was determined using the Folin–Ciocalteu reagent, with absorbance measured at 765 nm and a calibration curve based on gallic acid [30]. The total flavonoid content was analyzed using a sodium nitrite-aluminum chloride reaction, with absorbance read at 510 nm and a catechin calibration curve [31]. DPPH radical scavenging activity was assessed with a DPPH ethanol solution (6 × 10^−5^ mol/L), measuring absorbance at 525 nm and calibrating with Trolox [31]. Ferric ion reduction capacity (FRAP) was determined using TPTZ/iron chloride reagent, with absorbance measured at 595 nm and a calibration curve based on iron sulfate [32].

### 2.8. Statistical Analysis

The statistical analysis in this study was conducted using Microsoft^®^ Excel^®^ for Microsoft 365 MSO (Version 2412 Build 16.0.18324.20092 64-bit). The data were analyzed using One-way Analysis of Variance (ANOVA) to identify statistically significant differences between the samples. Post-hoc comparisons were performed using Tukey’s HSD (Honestly Significant Difference) test to pinpoint specific group differences. Statistical significance was determined at a threshold of *p* < 0.05, and results were presented with appropriate annotations in tables and figures to facilitate interpretation.

## 3. Results and Discussion

### 3.1. Macronutrients and Free Sugars

**Figure 3:**
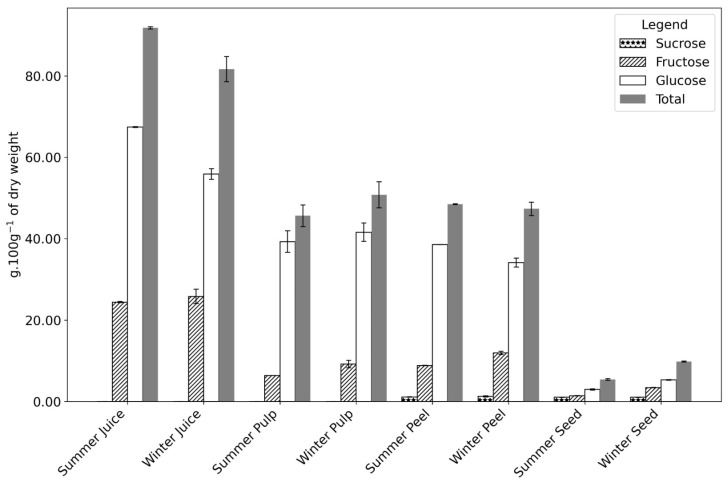
Sugar composition g·100 g^−1^ of the dry weight of *Opuntia* fruit parts (juice, pulp, peel, and seed) collected during summer and winter seasons. The components analyzed include fructose, glucose, sucrose, and total sugars. Numerical data can be found in Appendix A, Table A1.

The four parts of the fruit (juice, pulp, peel, and seed) exhibit typical compositions, with high moisture content followed by sugar (remaining carbohydrates), as shown in Table 1, Figure 3, and Table A1. The juice contains the least fiber, as fibrous components are removed during processing. The pulp has a higher fiber content due to the presence of seeds, while the peel’s high fiber concentration primarily serves as a physical barrier, providing structure and protection. The peel’s fiber content is also associated with higher ash levels, as ash is often chemically bound to fiber, resulting in a lower protein percentage. The composition of these fruit parts aligns with values reported in the literature. For example, ash levels of approximately 0.3 g·100 g^−1^ of dry weight have been reported in similar studies [8], consistent with the values observed for juice and pulp in this study (Table 1). Similar trends were noted for fat and moisture. Slight differences may arise due to variations in fruit age and growth conditions [33].

In the seed, higher moisture content was observed in winter (54.96 vs. 49.52 g·100 g^−1^ fw in summer), with no significant differences in fat or protein levels between seasons. However, the sugar content in winter seeds was double that of summer, driven primarily by a 2.4-fold increase in fructose, suggesting metabolite accumulation (Figure 3 and Table A1).

A much lower seed moisture value (6.9 ± 0.1 g·100 g^−1^ fw) has been reported in other studies [34] compared to this study’s results (49.52–54.96 g·100 g^−1^ fw). This discrepancy is likely due to differing sample preparations, as the seeds in their study were air-dried. Ash values were consistent with the literature, at 1.13 ± 0.02 g·100 g^−1^ dw [34] and 1.10 ± 0.10 g·100 g^−1^ dw [35]. The lipid content reported in the literature varies widely, from 11.75 ± 0.10 g·100 g^−1^ dw [35] to 3.66 ± 0.21 g·100 g^−1^ dw [36], with this study’s results falling between these values (Table 1). Protein levels were also similar to reported values: 5.40 ± 0.40 g·100 g^−1^ dw [35] and 4.48 ± 0.01 g·100 g^−1^ dw [36].

The seed fiber content in this study was significantly higher (80.20 ± 0.16 g·100 g^−1^ dw in summer and 73.98 ± 1.56 g·100 g^−1^ dw in winter) compared to values reported in the literature, such as 12.47 g·100 g^−1^ dw [37] and 45.8 g·100 g^−1^ dw [38]. These discrepancies may be due to variations in seed maturity, which influences lignification and fiber content, as well as differences in sampling, grinding, or sieving techniques. Genetic differences among *Opuntia* varieties and environmental factors like soil quality, water availability, and climate could further contribute to the observed variations. Plants grown under stress may allocate more resources to fiber production as an adaptive response.

Finally, different stages of fruit development can affect the composition, with ash levels typically decreasing and fat levels increasing as the fruit matures [38]. Additionally, the nitrogen content in betalain pigments, responsible for the fruit’s coloration, may artificially inflate protein values obtained through Kjeldahl analysis.

**Figure A1:**
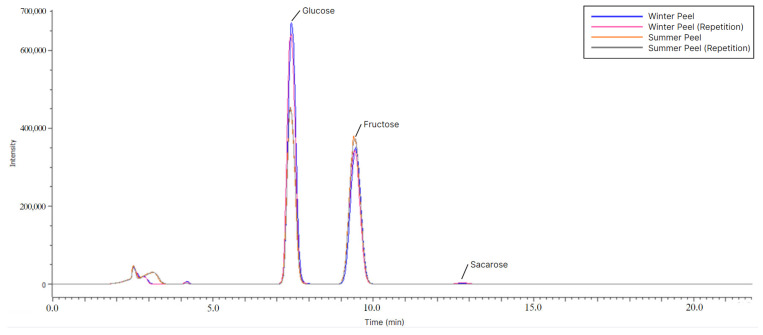
Chromatogram for the free sugar determination of the peel samples.

Sugar content is highest in the juice, as the juicing process removes the fibrous components, increasing the sugar amount. Andreu Nuncio-Jáuregui [39] reported that the peel constantly shows less sugar overall (in both glucose and fructose) than the pulp, as also indicated by our results. In all samples, a significantly higher amount of sugars (remaining carbohydrates) was registered in the winter samples. Figure 3, Table A1, and the chromatogram in Figure A1 show an accumulation of fructose in the winter compared to the summer sample. This carbohydrate is the main reason for the higher sugar content in winter. The role of fructose in a plant is not only as a source of energy but also as a regulatory metabolite.

Fructose plays a dual role in plants as both an energy source and a regulatory metabolite. Increased fructose levels in plants are strongly associated with cold resistance. Under cold stress, plants degrade starch into simpler sugars, such as glucose and fructose, to provide osmoprotection and sustain cellular functions [40,41]. Specifically, fructose and its derivatives, such as fructose-6-phosphate (F6P) and fructose-1,6-bisphosphate (FBP), contribute to maintaining homeostasis under low temperatures and exhibit significant radical scavenging properties.

In cucumbers, the CsSWEET2 transporter has been shown to enhance cold tolerance by increasing glucose and fructose levels, thereby reducing electrolyte leakage and improving resistance to cold stress [42]. Additionally, fructans, polymers of fructose synthesized from sucrose, stabilize cell membranes and reduce water loss during cold stress. This mechanism plays a critical role in protecting plants, as evidenced by an inverse correlation between fructan concentration and ambient temperature [43,44].

**Figure 4:**
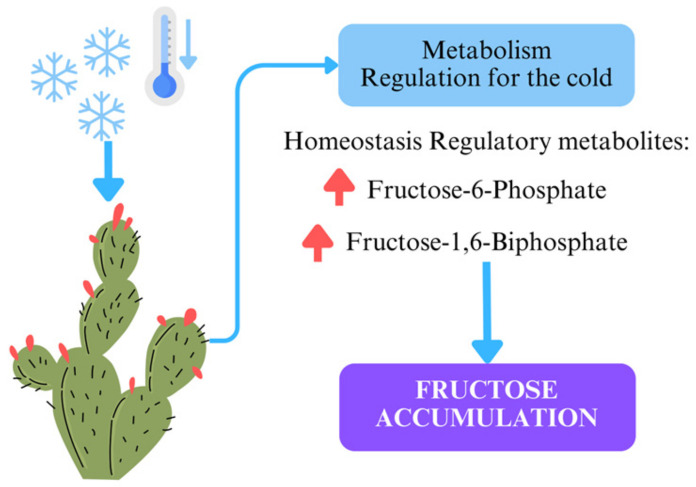
Illustration of metabolic regulation in *Opuntia ficus-indica* under cold stress. The decrease in temperature triggers the plant’s metabolic response, leading to the accumulation of regulatory metabolites such as fructose-6-phosphate and fructose-1,6-biphosphate. These metabolites aid in maintaining homeostasis and result in increased fructose accumulation, a key adaptation for cold resistance.

These processes explain the significant accumulation of fructose observed in the winter samples. This metabolic shift not only highlights the plant’s adaptation to cold stress but also positions *Opuntia* as an excellent candidate for natural sweeteners or energy-boosting products. Moreover, its high fiber content supports its classification as a functional food, promoting gut health. A diagram of this mechanism is presented in Figure 4. Further research into enzymatic activities and gene expression regulating these metabolic shifts could provide deeper insights into *Opuntia*’s adaptive strategies.

### 3.2. Antioxidants

**Figure 5:**
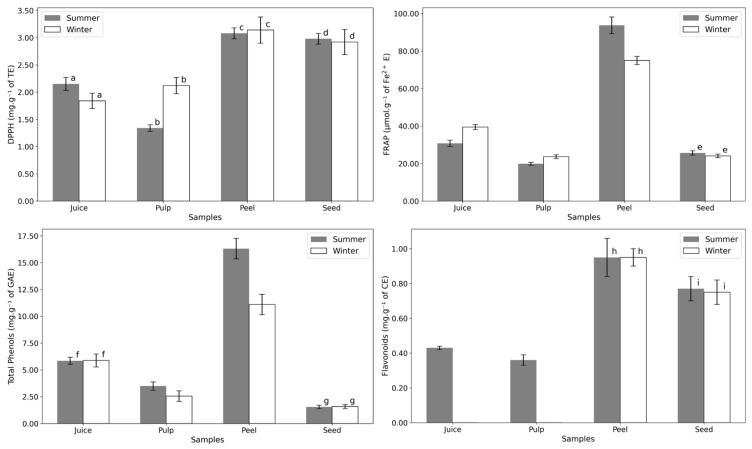
Antioxidant power essays of the different fruit parts in summer and winter: DPPH (mg·g^−1^ of Trolox equivalents dw), FRAP (µmol·g^−1^ of Fe^2+^ equivalents dw), Total Phenols (mg·g^−1^ of gallic acid equivalents dw), and Flavonoids (mg·g^−1^ of catechin equivalents dw). Letters indicate statistical comparisons: values with the same letter show no significant differences (*p* > 0.05) between summer and winter samples, as determined by a one-way ANOVA followed by a post-hoc Tukey HSD test. Numerical data can be found in Appendix A, Table A2.

The peel consistently shows the highest antioxidant power and bioactive compound content (Figure 5 and Table A2). This is expected, as the peel serves as the fruit’s outer protective layer, directly exposed to environmental stressors such as sunlight. All tests indicated no significant seasonal variation in the antioxidant potential of the seed. Interestingly, while flavonoids were consistently detected in summer samples, they were absent in the juice and pulp of winter samples. The lack of variation in DPPH results suggests resilience in *Opuntia* to seasonal environmental changes, making it a reliable year-round source of antioxidants.

Antioxidant activity typically increases under environmental stress, such as drought, excessive sunlight, low temperatures, or high soil salinity. Drought, in particular, is a major stress factor that influences plant growth and development [45]. This could explain the slight rise in antioxidant status in summer samples, likely due to higher flavonoid levels under water-limited conditions.

Dib and Seladji [46] reported a total phenolic content of 38.99 ± 0.10 mg·g^−1^ dw of Gallic Acid Equivalent (GAE) and a total flavonoid content of 31.58 ± 2.00 mg·g^−1^ dw of Catechin Equivalents (CE) for *Opuntia* seeds, values much higher than those found in this study. Gonzalez and Saucedo [47] reported phenolic contents of 4.45 ± 0.30 mg·g^−1^ dw of GAE for the peel, 1.68 ± 0.54 mg·g^−1^ dw of GAE for the pulp, and 2.13 ± 0.12 mg·g^−1^ dw of GAE for the juice of *Opuntia*. In comparison, the phenolic contents in this study were considerably higher for all parts of the fruit, with the peel consistently exhibiting the highest antioxidant values across all assays.

The same authors [47] conducted a DPPH inhibition assay, finding slightly higher antioxidant activity for the peel (4.96 ± 1.41 mg·g^−1^ dw of Trolox Equivalents (TE)) compared to this study’s value of 3.14 ± 0.24 mg·g^−1^ dw of TE. However, antioxidant power was higher in this study for the pulp and juice (e.g., 2.12 ± 0.15 mg·g^−1^ dw of TE vs. 0.60 ± 0.06 mg·g^−1^ dw of TE for the pulp and 2.15 ± 0.12 mg·g^−1^ dw of TE vs. 0.59 ± 0.06 mg·g^−1^ dw of TE for the juice).

Unfortunately, there are no reported studies on the winter fruit of *Opuntia ficus-indca* that measure antioxidant power, phenolic content, or flavonoid content. However, we can compare it to other fruit-bearing arid-soil preferring plants. *Citrullus colocynthis* is a desert viney plant that grows in sandy arid soils and bears a fruit known as Abu Jahl’s melon. Phenolic contents and antioxidant activity in *Citrullus colocynthis* fruits vary between seasons with the highest phenolics in rinds and highest antioxidant activities being recorded during summer [48], mirroring the general increase seen in *Opuntia* fruits in summer.

Typically, higher antioxidant power (as measured by DPPH or FRAP) correlates with increased phenolic or flavonoid content. However, this relationship does not always hold in the studied samples. For example, while the summer pulp contained higher phenolic content compared to the winter pulp, it exhibited a lower FRAP value. This discrepancy may be attributed to betalains, which are glucoside compounds that are the main antioxidants in *Opuntia*. Betalains are not detectable in phenol or flavonoid assays, yet they significantly contribute to the antioxidant activity of the fruit [1]. The same can be said for carotenoids, which are lipophilic pigments and potent antioxidants commonly found in *Opuntia* [1].

The correlation between bioactive compound content and antioxidant activity is evident in the results displayed in Figure 5. The peel, which consistently exhibited the highest levels of total phenolics and flavonoids, also demonstrated the greatest antioxidant potential across all assays (DPPH and FRAP). This aligns with the understanding that phenolic and flavonoid compounds are key contributors to antioxidant activity due to their ability to scavenge free radicals and chelate metals. However, the lack of seasonal variation in the seed’s antioxidant potential, despite differences in flavonoid levels, suggests that other compounds, such as betalains or tocopherols, may play a significant role in maintaining consistent antioxidant activity. These findings highlight the complex interplay of bioactive compounds in determining the antioxidant capacity of *Opuntia ficus-indica* fruit parts.

The apparent absence of flavonoids in the winter juice and pulp, despite their presence in the seed and peel, can be attributed to several biological and methodological factors. Flavonoids are known to be sensitive to environmental factors such as pH, temperature, and enzymatic activity. The pulp and juice, being more aqueous and metabolically active, may experience higher enzymatic degradation of flavonoids during winter. In contrast, the seed and peel, which serve as protective tissues, are better equipped to retain these compounds under stressful conditions.

Additionally, flavonoid biosynthesis is highly tissue-specific and regulated by environmental cues. The peel is directly exposed to environmental stressors, such as UV radiation and cold, which stimulate flavonoid production as a protective mechanism. The juice and pulp, being internal tissues, do not experience the same level of direct environmental exposure, leading to lower flavonoid accumulation in these parts.

It is also important to consider the limitations of the analytical method used. If the flavonoid concentrations in the juice and pulp are particularly low in winter, they may fall below the detection threshold of the assay, resulting in an apparent absence.

### 3.3. Vitamin E

Vitamin E, a vital nutrient in the human diet, encompasses a group of tocopherols and tocotrienols, with α-tocopherol exhibiting the highest biological activity [49]. These compounds possess strong antioxidant properties and are linked to preventing chronic diseases related to oxidative stress. Vitamin E is also widely used in cosmetics for its ability to prevent product oxidation, extend shelf life, and protect skin against oxidative damage. *Opuntia* oil, known for its safety, is gaining popularity in cosmetic formulations for its moisturizing properties and its potential to reduce redness and inflammation [50].

**Figure 6:**
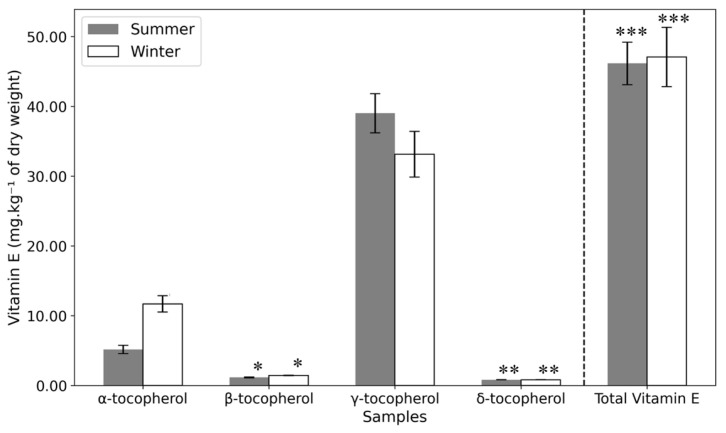
Vitamin E content of the seeds of summer and winter samples (mg·kg^−1^ of dry weight). Statistical significance is indicated by asterisks (*, **, ***), with *p*-values less than 0.05, as determined by a one-way ANOVA followed by a post-hoc Tukey HSD test.

In this study, α-, β-, γ-, and δ-tocopherol were identified in the samples (Table 2 and Figure 6). While α-tocopherol was distributed throughout the fruit and seed, γ-tocopherol was predominant in the seed. Notably, α-tocopherol levels were significantly higher in winter samples compared to summer. This aligns with findings in seagrass (*Cymodocea nodosa*), where increased α-tocopherol has been associated with enhanced cold resistance during winter [51]. Cold stress has been shown to trigger the expression of genes involved in α-Tocopherol biosynthesis, such as gamma-tocopherol methyl transferase (γ-TMT), enhancing the plant’s antioxidative capacity, preserving the structural integrity of cell membranes, and maintaining overall cell integrity [35,52]. This is because α-Tocopherol works in conjunction with other antioxidants to mitigate oxidative damage [53]. The role of α-Tocopherol against cold stress is further demonstrated by tocopherol-deficient mutants that show cold stress-caused impairment on cell wall development in phloem transfer cells, indicating the necessity of α-Toc for proper phloem loading and cold adaptation [54]. This may explain the observed trends in *Opuntia*. Further research into enzymatic activities and gene expression governing these metabolic shifts could provide deeper insights into the plant’s adaptive mechanisms.

In a literature review [52], it was reported that *Opuntia* seed oil with γ-tocopherol as the dominant form (67.5–98.5% of total vitamin E), followed by δ-tocopherol (0.46–8.26%) and α-tocopherol (0.11–1.95%). These findings align with the present study, except for the notably higher α-tocopherol content in the samples analyzed here. Differences in extraction methods could explain this variation, as the Soxhlet method used in some studies involves higher temperatures that may degrade vitamin E. γ-tocopherol is constantly reported as the predominant form of Vitamin E in *Opuntia ficus-indica* (94.12% [35] and 92%, respectively [55]), with δ-tocopherol (6.24%) and α-tocopherol (1.33%) present in smaller amounts [35]. These studies focused on plants from Tunisia, Morocco, Algeria, and Turkey, regions with higher average temperatures than Portugal. If α-tocopherol levels are indeed higher in plants exposed to colder climates, this could explain the elevated levels in the Portuguese samples. This represents a nutritional advantage, as α-tocopherol is the most biologically active form of vitamin E.

The antioxidant potential of vitamin E forms varies due to differences in their chemical structures, which affect their ability to donate electrons or hydrogen atoms [49]. Alpha-tocopherol generally exhibits the highest antioxidant power due to its fully methylated chromanol ring, stabilizing its structure and optimizing hydrogen donation [49]. However, this variation does not appear to influence the antioxidant test results for the summer and winter samples (Figure 5 and Table A2).

The consistently high vitamin E content in *Opuntia* across both seasons highlights its value as an ingredient in anti-aging creams, sunscreens, and moisturizers. Its strong antioxidant properties and ability to reduce oxidative stress make *Opuntia* seed oil a promising candidate for cosmetic formulations [56].

### 3.4. Fatty Acids

Fat is a minor component of *Opuntia*, with seeds containing the highest fat content. Seeds, which make up 30–40% of the fruit’s dry weight [57], are often discarded as waste after pulp extraction. Millions of kilograms of fruit seeds are discarded annually, posing environmental concerns. However, proper utilization of this waste could create a valuable source of oil and meal [34], making it essential to study the oil composition to explore its potential uses and benefits.

The primary fatty acid in the samples was linoleic acid (C18:2n6c), accounting for 57.39–59.26% (Table 3). Linoleic acid, a polyunsaturated omega-6 essential fatty acid, must be obtained through the human diet and is widely recognized for its health benefits. Previous studies confirm its dominance in *Opuntia* oil, with reported values ranging from 53.84% to 70.3% of total fatty acids [35,36,37,38,39,46]. Its high linoleic acid content supports the increasing application of *Opuntia* oil in cosmetics, offering anti-inflammatory properties, acne reduction, skin-lightening, and moisture retention benefits [52]. Additionally, the consistent linoleic acid levels across seasons ensure reliability in cosmetic formulations.

Oleic acid (C18:1n9c) was the second most abundant fatty acid (17.61–18.09%), followed by palmitic acid (C16:0) at 12.24–12.86% (Table 3). These values align with the reported ranges of 15.2–25.52% for oleic acid and 9.32–14.06% for palmitic acid [35,37,38,58,59]. Vaccenic acid (C18:1n7) was also detected at 5.71–6.87%, consistent with the 5.0–5.5% reported in the literature [34,35,37,38,57,58,59,60]. Interestingly, vaccenic acid has been found only in green and orange prickly pear varieties in previous studies, which aligns with this study’s focus on the orange variety [58].

Winter samples showed slightly higher saturation, with the primary differences between seasons observed in minor lipid components, including α-linolenic acid (C18:3n3) and eicosanoic acid (C20:1n9). Other fatty acids, such as arachidic acid (C20:0), were found in trace amounts (0.23–0.25%), comparable to literature values (0.42–0.66%) [38] and (0.30%) [34].

The fatty acid profile of *Opuntia* seed oil, dominated by linoleic acid, exhibited remarkable stability across seasons. This resilience suggests an inherent ability of *Opuntia ficus-indica* to maintain its bioactive and nutritional properties despite environmental changes, offering unique advantages for sustainable production. Compared to other plant species that display significant seasonal fluctuations, *Opuntia*’s stable fatty acid composition (~57–59% linoleic acid) makes it ideal for industries requiring consistent lipid profiles, such as cosmetics and nutrition.

## 4. Conclusions

Winter and summer samples show a typical fruit composition, with their main components being water, sugar, and fiber. This study highlights the significant seasonal variations in the nutritional and chemical profiles of *Opuntia* fruits. Winter fruits exhibited higher fructose levels, with the seed containing 2.4 times more fructose and 1.8 times more total sugars than summer seeds. The pulp also showed an increase in sugar content, rising from 45.65% in summer to 50.78% in winter. Additionally, winter samples demonstrated increased moisture content, with seed moisture increasing from 49.52% to 54.96%, and a notable rise in α-tocopherol levels, which were 2.3 times higher in winter (from 5.17 mg/kg to 11.69 mg/kg). These findings suggest metabolic adaptations in the plant to colder conditions, such as the accumulation of fructose to enhance cold resistance.

The peel consistently showed the highest antioxidant activity and bioactive compound content, with total phenols measuring 16.31 mg/g GAE in summer and 11.10 mg/g GAE in winter. Antioxidant activity in the peel, measured through DPPH, showed minimal seasonal variation (3.08 mg/g TE in summer vs. 3.14 mg/g TE in winter). However, overall antioxidant power was higher in summer, likely due to environmental stressors like drought and intense sunlight.

Fatty acid analysis confirmed linoleic acid as the dominant component in seed oil across seasons, constituting 59.26% in summer and 57.39% in winter. A slight increase in saturation was observed in winter, with total saturated fatty acids rising from 16.43% to 17.41%.

Overall, this study underscores the importance of *Opuntia* as a sustainable crop with distinct seasonal profiles. Its ability to produce fruits in both summer and winter demonstrates its potential as a sustainable crop in regions with extreme weather conditions. Its bioactive-rich composition, particularly the higher α-tocopherol content in winter and enhanced antioxidant potential in summer, highlights its potential applications in functional foods, cosmetics, and cultivation in colder regions.

## Figures and Tables

**Table 1:** Macronutrient, ash, and moisture composition of various fruit parts (juice, pulp, peel, and seed) of the collected samples (summer, winter) (g·100 g^−1^ of dry weight ± SD).

Sample		Moisture *	Ash	Lipids	Protein	Total Fiber	Carbohydrates **
Juice	Summer	88.44 ± 0.06 *^a^*	0.35 ± 0.02*^b^*	0.36 ± 0.02 *^c^*	3.26 ± 0.06 *^d^*	2.50 ± 0.11	93.53 ± 0.27
	Winter	88.80 ± 0.16 *^a^*	0.37 ± 0.01 *^b^*	0.40 ± 0.02 *^c^*	3.39 ± 0.29 *^d^*	3.74 ± 0.06	92.10± 0.38
Pulp	Summer	82.26 ± 0.63	0.27 ± 0.03	0.66 ± 0.05 *^e^*	3.48 ± 0.04*^f^*	32.19 ± 2.02 *^g^*	63.40 ± 2.10
	Winter	84.95 ± 0.91	0.41 ± 0.03	0.53 ± 0.04 *^e^*	3.96 ± 0.40 *^f^*	32.68 ± 1.06 *^g^*	62.42± 1.09
Peel	Summer	84.56 ± 0.43	1.51 ± 0.07 *^h^*	0.94 ± 0.01 *^i^*	2.92 ± 0.15*^j^*	26.18 ± 0.48	68.45 ± 0.71
	Winter	86.48 ± 0.29	1.76 ± 0.11 *^h^*	0.81 ± 0.01 *^i^*	2.96 ± 0.20 *^j^*	18.31 ± 0.24	76.16 ± 0.32
Seed	Summer	49.52 ± 1.41	1.77 ± 0.11	6.14 ± 0.34 *^k^*	6.83 ± 0.25 ^*l*^	80.20 ± 0.16	5.06 ± 0.86
	Winter	54.96 ± 1.01	2.22 ± 0.01	7.07 ± 0.10 *^k^*	6.72 ± 0.00 *^l^*	73.98 ± 1.56	10.01 ± 1.67

* in g·100 g^−1^ ± SD fresh weight; ** Carbohydrates were calculated by subtracting the fat, protein, total fiber, and ash from the total of 100 g·100 g^−1^; Each column’s letters indicate statistical comparisons between the summer and winter samples for the same parameter. Values that share the same letter within a column are not significantly different (*p* > 0.05), as determined by a one-way ANOVA followed by a post-hoc Tukey HSD test.

**Table 2:** Vitamin E content of the seeds of summer and winter samples (mg·kg−1 of dry weight ± SD).

Vitamin E (mg·kg^−1^ of Dry Weight)	Samples	
	Summer	Winter
α-tocopherol	5.17 ± 0.59	11.69 ± 1.16
β-tocopherol	1.16 ± 0.08 *^a^*	1.43 ± 0.03 *^a^*
γ-tocopherol	39.02 ± 2.82	33.15 ± 3.29
δ-tocopherol	0.82 ± 0.03 *^b^*	0.82 ± 0.02 *^b^*
Total	46.17 ± 3.04 *^c^*	47.09 ± 4.26 *^c^*

Within each row, the same letter represents no significant differences (*p* > 0.05) between collected samples (Summer and Winter) determined by one-way ANOVA with a post-hoc Tukey HSD Test.

**Table 3:** Fatty acid content of the seeds of the summer and winter samples (total fatty acid % ± SD).

Compound Name	Summer	Winter
Myristic Acid (C14:0)	0.06 ± 0.01 *^a^*	0.10 ± 0.00 *^a^*
Palmitic Acid (C16:0)	12.24 ± 0.06 *^b^*	12.86 ± 0.34 *^b^*
Palmitoleic Acid (C16:1)	0.67 ± 0.03 *^c^*	0.81 ± 0.02 *^c^*
Stearic Acid (C18:0)	3.59 ± 0.19 *^d^*	3.77 ± 0.23 *^d^*
Oleic Acid (C18:1n9c)	17.61 ± 0.40 *^e^*	18.09 ± 1.80 *^e^*
Vaccenic Acid (C18:1n7) *	5.71 ± 0.13 *^f^*	5.90 ± 0.23 *^f^*
Linoleic acid (C18:2n6c)	59.26 ± 0.21 *^g^*	57.39 ± 1.73 *^g^*
Arachidic Acid (C20:0)	0.23 ± 0.01 *^h^*	0.25 ± 0.02 *^h^*
α-Linolenic Acid (C18:3n3)	0.17 ± 0.01	0.24 ± 0.02
Eicosanoic Acid (C20:1n9)	0.14 ± 0.01	0.17 ± 0.00
Behenic Acid (C22:0)	0.18 ± 0.01 *^i^*	0.18 ± 0.01 *^i^*
Lignoceric Acid (C24:0)	0.11 ± 0.01 *^j^*	0.25 ± 0.02 *^j^*
Total SFA	16.43 ± 0.28	17.41 ± 0.63
Total MUFA	24.14 ± 0.57	24.97 ± 2.06
Total PUFA	59.44 ± 0.22	57.62 ± 1.75
Total UFA	83.58 ± 0.79	82.59 ± 3.81

* Identified as vaccenic acid according to bibliography [31,33,34,48,49,50,51,52]. Within each row, the same letter represents no significant differences (*p* > 0.05) between collected samples (summer and winter) determined by one-way ANOVA with a post-hoc Tukey HSD Test.

## Data Availability

All generated data are included in the article and in its respective Appendix A.

## Appendix A

**Table A1:** Sugar composition of various fruit parts (juice, pulp, and peel) of the collected samples (watered, summer 2019, winter 2019, and summer 2020) (% (m/m) of dry weight ± SD).

Samples		Fructose	Glucose	Sucrose	Total
Juice	Summer	24.40 ± 0.15 ^a^	67.43 ± 0.13	n.d.	91.83 ± 0.28
	Winter	25.82 ± 1.76 ^a^	55.85 ± 1.31	n.d.	81.67 ± 3.07
Pulp	Summer	6.38 ± 0.01	39.27 ± 2.65	n.d.	45.65 ± 2.66
	Winter	9.21 ± 0.91	41.57 ± 2.27	n.d.	50.78 ± 3.18
Peel	Summer	8.86 ± 0.05	38.51 ± 0.02	1.11 ± 0.04	48.48 ± 0.11
	Winter	11.95 ± 0.39	34.10 ± 1.09	1.27 ± 0.15	47.32 ± 1.63
Seed	Summer	1.43 ± 0.06	2.96 ± 0.15	1.05 ± 0.02 ^b^	5.44 ± 0.21
	Winter	3.45 ± 0.05	5.32 ± 0.07	1.05 ± 0.02 ^b^	9.82 ± 0.14

n.d. not detected. Within each column, the same letter represents no significant differences (*p* > 0.05) between collected samples (summer and winter) determined by one-way ANOVA with a post-hoc Tukey HSD Test.

**Table A2:** Antioxidant power and bioactive compounds in the collected samples’ dry weight (ethanol/water extract) of various fruit parts (juice, pulp, and peel and seed).

		DPPH	FRAP	Total Phenols	Flavonoids
Samples		mg·g^−1^ of TE	µmol·g^−1^ of Fe^2+^ E	mg·g^−1^ of GAE	mg·g^−1^ of CE
Juice	Summer	2.15 ± 0.12 ^a^	30.77 ± 1.68	5.85 ± 0.33 ^f^	0.43 ± 0.01
	Winter	1.84 ± 0.14 ^a^	39.49 ± 1.36	5.88 ± 0.60 ^f^	n.d.
Pulp	Summer	1.34 ± 0.06 ^b^	19.88 ± 0.81	3.49 ± 0.39	0.36 ± 0.03
	Winter	2.12 ± 0.15 ^b^	23.66 ± 0.99	2.56 ± 0.49	n.d.
Peel	Summer	3.08 ± 0.10 ^c^	93.72 ± 4.47	16.31 ± 0.96	0.95 ± 0.11 ^h^
	Winter	3.14 ± 0.24 ^c^	74.96 ± 2.20	11.10 ± 0.95	0.95 ± 0.05 ^h^
Seed	Summer	2.98 ± 0.10 ^d^	25.64 ± 1.13 ^e^	1.55 ± 0.16 ^g^	0.77 ± 0.07 ^i^
	Winter	2.92 ± 0.23 ^d^	24.10 ± 0.99 ^e^	1.58 ± 0.19 ^g^	0.75 ± 0.07 ^i^

n.d. not detected. Within each column, the same letter represents no significant differences (*p* > 0.05) between collected samples (summer and winter) determined by one-way ANOVA with a post-hoc Tukey HSD Test.
